# The Frequency of Intrathyroidal Follicular Helper T Cells Varies with the Progression of Graves' Disease and Hashimoto's Thyroiditis

**DOI:** 10.1155/2022/4075522

**Published:** 2022-02-02

**Authors:** Yun Cai, Zhixiao Wang, Xiaoyun Liu, Ling Wei, Shushu Li, Xuqin Zheng, Tao Yang, Xinyu Xu

**Affiliations:** Department of Endocrinology, The First Affiliated Hospital of Nanjing Medical University, Nanjing, Jiangsu Province, China

## Abstract

**Objective:**

Autoimmune thyroid diseases (AITD), mainly Graves' disease (GD) and Hashimoto's thyroiditis (HT), are common organ-specific autoimmune diseases characterized by circulating antibodies and lymphocyte infiltration. Follicular helper T (Tfh) cell dysregulation is involved in the development of autoimmune pathologies. We aimed to explore the role of intrathyroidal and circulating Tfh cells in patients with GD and HT.

**Methods:**

Ultrasound-guided thyroid fine-needle aspiration (FNA) was conducted in 35 patients with GD, 40 patients with HT, and 22 patients with nonautoimmune thyroid disease (nAITD). Peripheral blood samples were also obtained from 40 patients with GD, 40 patients with HT, and 40 healthy controls. The frequencies of intrathyroidal and circulating Tfh cells from FNA and peripheral blood samples were assessed by flow cytometry. Additionally, the correlations between the frequencies of the Tfh cells and the levels of autoantibodies and hormones or disease duration were analyzed.

**Results:**

The frequency of intrathyroidal CD4^+^CXCR5^+^ICOS^high^ Tfh cells was higher in HT patients than in GD patients. Significant correlations were identified between the percentages of circulating and intrathyroidal Tfh cells and the serum concentrations of thyroid autoantibodies, especially thyroglobulin antibodies (TgAb), in AITD. Intrathyroidal CD4^+^CXCR5^+^ICOS^high^ Tfh cells were positively correlated with free triiodothyronine (FT3) in HT patients but negatively correlated with FT3 in GD patients. In addition, HT patients with a longer disease duration had an increased frequency of intrathyroidal CD4^+^CXCR5^+^ICOS^high^ and CD4^+^CXCR5^+^PD-1^+^ Tfh cells. In contrast, in the GD patients, a longer disease duration did not affect the frequency of intrathyroidal CD4^+^CXCR5^+^ICOS^high^ but was associated with a lower frequency of CD4^+^CXCR5^+^PD-1^+^ Tfh cells.

**Conclusions:**

Our data suggest that intrathyroidal Tfh cells might play a role in the pathogenesis of AITD and they are potential immunobiomarkers for AITD.

## 1. Introduction

Graves' disease (GD) and Hashimoto's thyroiditis (HT) are autoimmune thyroid diseases (AITD) that are characterized by the accumulation of B and T lymphocytes in the thyroid gland and the production of autoantibodies targeting the thyroid [1–3]. Both GD and HT share the immunologic manifestation of the presence of circulating autoantibodies such as thyrotropin receptor autoantibodies (TRAb), thyroid peroxidase autoantibodies (TPOAb), and thyroglobulin autoantibodies (TGAb) [4]. The clinical hallmarks of GD and HT are thyrotoxicosis and hypothyroidism, respectively. However, the immune mechanisms involved in the cellular- and humoral-mediated pathogenesis of AITD are not completely understood.

Follicular helper T (Tfh) cells are a specialized CD4^+^ T cell subset associated with helping B cells produce antibodies while confronting antigenic challenges [5]. Tfh cells are characterized by the surface expression of CXC chemokine receptor 5 (CXCR5), inducible costimulator (ICOS), and programmed death-1 (PD-1); the expression of the transcription factor B cell lymphoma-6 (Bcl-6); and high-level IL-21 secretion [6–9]. Tfh cells have been well investigated in many autoimmune diseases, such as systemic lupus erythematosus (SLE), rheumatoid arthritis (RA), and Sjögren's syndrome (SS) [7, 10–12]. Aberrant expansion of Tfh cells can contribute to the production of pathogenic autoantibodies, which play an important role in the promotion of autoimmune diseases [13–15]. Recently, cancer immunotherapy utilizing immune checkpoint inhibitors (ICIs) against PD-1 or its ligand PD-L1 has been frequently accompanied by immune-related adverse events (irAEs), among which thyroid autoimmunity represents the most common endocrine side effect [16, 17]. The association between immunotherapy with anti-PD-1/PD-L1 antibodies and the development of thyroid disorders suggests that abnormal Tfh cells might be involved in the pathogenesis of AITD [17].

Although several studies have shown that circulating Tfh cells could be involved in the pathogenesis of GD or HT, the differences between the Tfh populations from peripheral blood and thyroid tissue and their role in AITD remain unclear [18–20]. Therefore, we investigated the frequencies of Tfh cells obtained from both peripheral blood and thyroid fine-needle aspiration (FNA) in GD and HT patients and aimed to explore the role of intrathyroidal and circulating Tfh cells in patients with AITD.

## 2. Materials and Methods

### 2.1. Subjects and Samples

#### 2.1.1. Peripheral Blood Samples from AITD Patients and Healthy Controls

Eighty patients with AITD, including 40 with GD and 40 with HT, were recruited. Autoimmune thyroid diseases were diagnosed clinically by endocrinologists and confirmed by clinical symptoms, abnormal levels of thyroid hormones, and autoantibodies to TRAb, TPOAb, and/or TgAb together with ultrasound examination. Seventeen patients (42.5%) with GD were treated with methimazole according to the titration method, and 20 patients (50.0%) with HT were treated with replacement therapy with levothyroxine. Forty age- and sex-matched healthy volunteers who did not have a medical history of thyroid or other autoimmune diseases were included as healthy controls. Peripheral blood samples were collected from all patients and healthy controls. The main clinical characteristics of these subjects are shown in Table 1.

#### 2.1.2. Thyroid FNA Samples from Patients with AITD and Nonautoimmune Thyroid Diseases (nAITD)

Seventy-five patients with AITD, namely, 35 with GD and 40 with HT, were included in this study. Eighteen patients (51.4%) with GD were treated with antithyroid drugs (15 patients with methimazole and 3 patients with propylthiouracil) according to the titration method, and 10 patients (25.0%) with HT were treated with replacement therapy with levothyroxine. Twenty-two subjects with thyroid diseases of nonautoimmune origin (nAITD), namely, thyroid nodules, were also recruited in the thyroid FNA control group from our outpatient endocrinology department. Patients with nAITD were euthyroid, tested negative for all three thyroid antibodies, and did not have a history of other autoimmune diseases. The baseline characteristics of the FNA subjects are shown in Table 2.

All subjects underwent ultrasonography (US) evaluation, and the examinations were performed by the same radiologist (L.W.) using linear-array sonographic scanners (6-18 MHz, Esaote MYLAB 60 system, Italy). The FNA was performed by a radiologist (L.W.) and another endocrinologist (Y.C., Z.W., or X.L.) under direct US guidance. We performed free-hand FNA using one pass of a 25-gauge needle (0.5 mm × 38 mm, Becton Dickinson) attached to a 5 mL syringe (Becton Dickinson) [21]. The transducer was placed over the hypoechogenic area of AITD subjects or a paranodule normal echogenic area of control subjects, and thyroid vascularity was avoided by color-flow Doppler examination. The needle was introduced perpendicular to the transducer, and then, the aspiration was performed. The needle was then rinsed in a tube containing 500 *μ*L of phosphate-buffered saline (PBS). During the procedure, all needle movements were continuously visualized by US in real time.

Additionally, peripheral blood samples and FNA samples were both obtained from 7 patients with GD (4 females and 3 males; aged 26 (34, 53) years) and 7 patients with HT (6 females and 1 male; aged 45 (41, 47) years).

Ethical approval for the research (including the consent procedure) was granted by the Human Ethics Committee of the First Affiliated Hospital of Nanjing Medical University (2019-SR-059), and informed consent was obtained from each participant.

### 2.2. Thyroid Function and Antithyroid Antibodies

Free triiodothyronine (FT3), free tetraiodothyronine (FT4), thyroid-stimulating hormone (TSH), TgAb, and TPOAb were all measured by chemiluminescence assays (Roche Diagnostics GmbH), while TRAb was tested by RIA (Cisbio Bioassays, France). Reference ranges for adults are TSH, 0.270–4.20 mIU/L; FT3, 3.10–6.80 pmol/L; FT4, 12.0–22.0 pmol/L; TgAb, <115 IU/mL; TPOAb, <34.0 IU/mL; and TRAb, 0-1.50 IU/L [22].

### 2.3. Flow Cytometric Analysis

Human peripheral blood mononuclear cells (PBMCs) were collected in heparin-coated tubes and isolated in Lymphoprep™ (Nycomed, Pharma AS, Oslo, Norway) gradients according to the manufacturer's protocol. Red blood cells in the thyroid FNA samples were lysed with RBC lysis/fixation solution (BioLegend, San Diego, CA). Then, the samples were washed with PBS and immunostained with the indicated antibodies. The following antibodies were used: FITC-mouse-anti-human CD4, PerCP-Cy5.5-anti-human CD185 (CXCR5), APC-anti-human CD278 (ICOS), and PE-anti-human CD279 (PD-1) (BioLegend, San Diego, CA). The stained cells were washed with PBS and analyzed by multiparameter flow cytometry (BD FACSCalibur™).

### 2.4. Statistical Analysis

Statistical analysis was performed using GraphPad Prism 9 software (GraphPad, San Diego, CA). As the baseline characteristics of the subjects were not normally distributed, the data were described using medians with quartiles (P25 and P75), and the statistical analyses were performed using nonparametric tests. Mann–Whitney tests were used to compare two sets of data. Kruskal-Wallis tests were used to compare three sets of data. The differences between classified variables were tested using chi-squared tests or Fisher's exact test. Correlation analyses were performed using the Spearman correlation method. *P* < 0.05 was considered to be statistically significant.

## 3. Results

### 3.1. Identification of Circulating and Intrathyroidal Tfh Cell Subsets in AITD Patients

**Figure 1:**
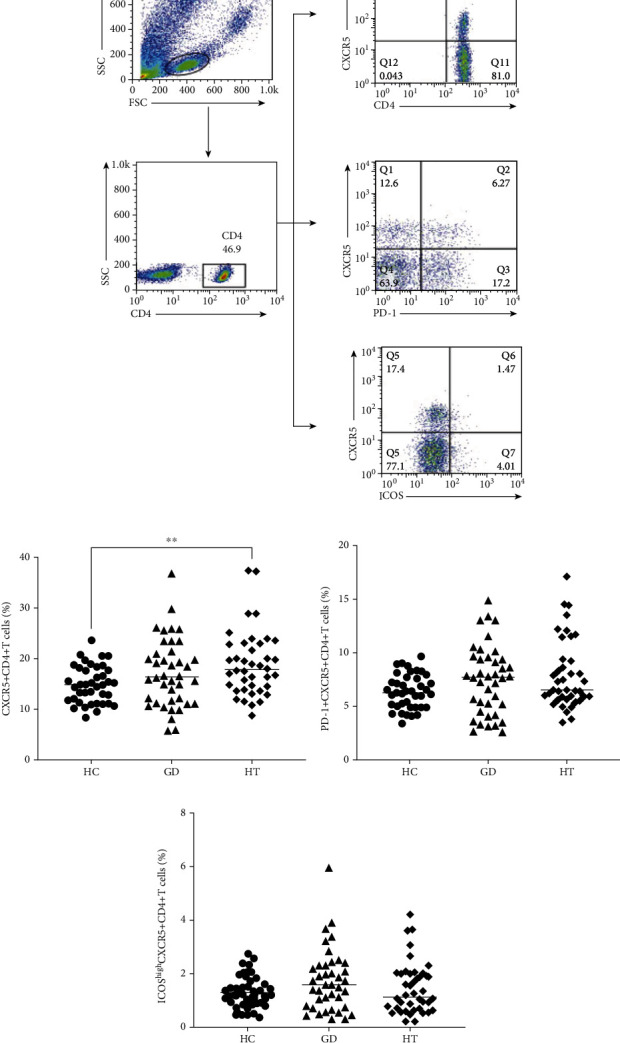
The frequencies of CD4^+^CXCR5^+^, CD4^+^CXCR5^+^PD-1^+^, and CD4^+^CXCR5^+^ICOS^high^ Tfh cells in the peripheral blood of GD and HT patients. (a) Representative dot plot showing the gating strategy for Tfh cells gated on CD4^+^ T cells. (b–d) The frequencies of CD4^+^CXCR5^+^, CD4^+^CXCR5^+^PD-1^+^, and CD4^+^CXCR5^+^ICOS^high^ Tfh cells in patients with GD (*n* = 40), HT (*n* = 40), and healthy controls (*n* = 40).

Tfh cells express high levels of CXCR5, PD-1, and ICOS. First, we gated CD4^+^ T cells in PBMCs and identified CXCR5^+^ T cells to distinguish Tfh cells from activated T cells in the peripheral blood. Then, we characterized the frequencies of CD4^+^CXCR5^+^ICOS^high^ and CD4^+^CXCR5^+^PD-1^+^ Tfh cells in these subjects by flow cytometric analysis (Figure 1(a)). The frequency of CD4^+^CXCR5^+^ T cells was significantly increased in HT patients compared with healthy controls (*P* = 0.0092) (Figure 1(b)), whereas the frequencies of CD4^+^CXCR5^+^PD-1^+^ and CD4^+^CXCR5^+^ICOS^high^ Tfh cells were comparable among these groups (Figures 1(c) and 1(d)).

**Figure 2:**
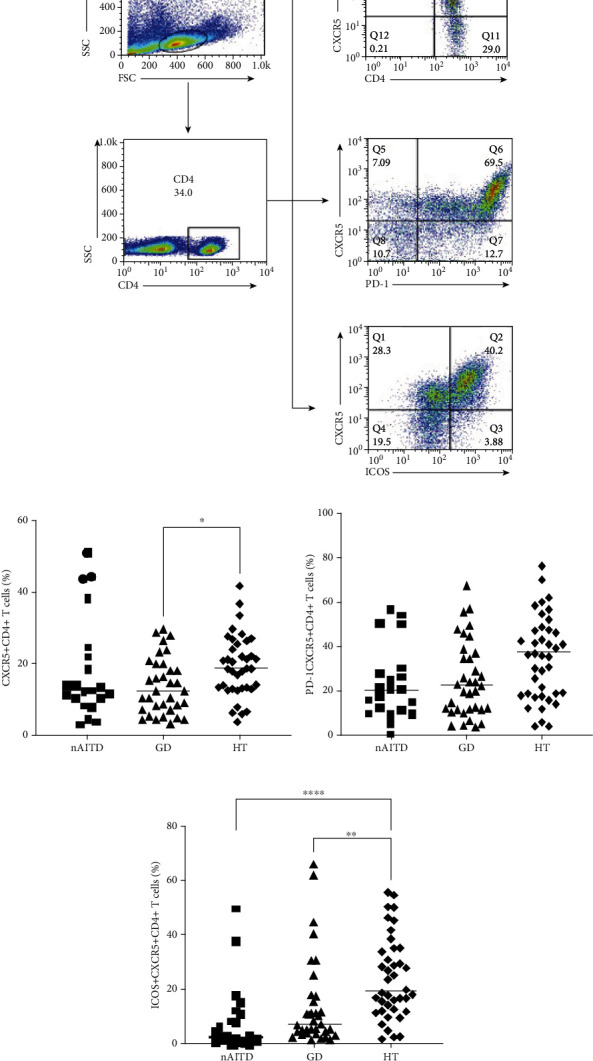
The frequencies of CD4^+^CXCR5^+^, CD4^+^CXCR5^+^PD-1^+^, and CD4^+^CXCR5^+^ICOS^high^ Tfh cells in the thyroid of GD and HT patients. (a) Representative dot plot showing the gating strategy for Tfh cells gated on CD4^+^ T cells. (b–d) The frequencies of CD4^+^CXCR5^+^, CD4^+^CXCR5^+^PD-1^+^, and CD4^+^CXCR5^+^ICOS^high^ Tfh cells in patients with GD (*n* = 35), HT (*n* = 40), and nAITD (*n* = 22).

As the frequency of Tfh cells was not significantly altered in the peripheral circulation, we then investigated intrathyroidal Tfh cell subsets (Figure 2(a)). The frequency of intrathyroidal CD4^+^CXCR5^+^ Tfh cells was higher in HT patients than in GD patients (*P* = 0.0290) (Figure 2(b)), while CD4^+^CXCR5^+^PD-1^+^ Tfh cells were not significantly different between the two groups (Figure 2(c)). Patients with HT had a higher frequency of CD4^+^CXCR5^+^ICOS^high^ Tfh cells than those with GD or the nAITD controls (*P* = 0.0031, *P* < 0.0001) (Figure 2(d)).

### 3.2. The Frequency of Circulating and Intrathyroidal CD4^+^CXCR5^+^ T Cells Is Associated with the Levels of TgAb in GD Patients

The presence of the stimulating TSHR antibody is a hallmark of GD. TPOAb is also a critical antibody, with approximately 80% of GD patients testing positive for this parameter [23]. Here, we showed that the frequencies of CD4^+^CXCR5^+^ T cells in both circulating and thyroid tissue were only positively correlated with the serum TgAb level in GD patients (Supplementary Figures 1A and 2A). However, no significant difference between the peripheral or intrathyroidal frequencies of CD4^+^CXCR5^+^ T cells and thyroid hormones in GD patients was observed (Supplementary Figures 1B and 2B).

The presence of circulating TPOAb usually accompanies positive TgAb in HT patients. The detection of either autoantibody in serum essentially indicates the presence of thyroid lymphocytic infiltration, and these two autoantibodies have also been used for the diagnosis of HT [4, 24]. We found that the frequency of CD4^+^CXCR5^+^ T cells in the peripheral circulation was positively correlated with TPOAb but negatively correlated with FT3 (Supplementary Figures 1C and 1D). Within the thyroid, the frequency of CD4^+^CXCR5^+^ T cells was only positively correlated with TgAb (Supplementary Figure 2C). We did not observe a significant correlation between intrathyroidal CD4^+^CXCR5^+^ Tfh cells and thyroid hormones in HT patients (Supplementary Figure 2D).

### 3.3. The Frequency of Tfh Cells Is Associated with the Levels of TgAb and FT3 in GD Patients

**Figure 3:**
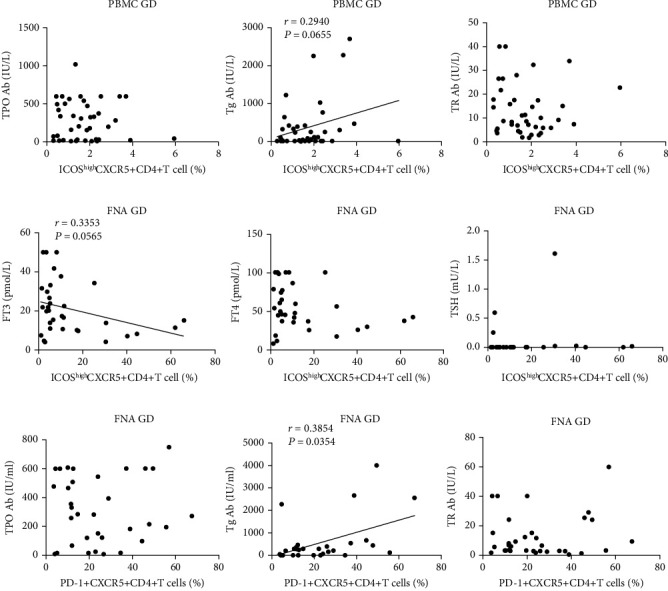
Correlations between Tfh cells and autoantibodies or thyroid function in GD patients. (a) Correlation between the frequency of circulating CD4^+^CXCR5^+^ICOS^high^ Tfh cells and the levels of autoantibodies in GD patients. (b) Correlation between the frequency of intrathyroidal CD4^+^CXCR5^+^ICOS^high^ Tfh cells and the levels of FT3, FT4, and TSH in GD patients. (c) Correlation between the frequency of intrathyroidal CD4^+^CXCR5^+^PD-1^+^ Tfh cells and the levels of autoantibodies in GD patients.

In GD patients, we investigated the relationships between Tfh cells and thyroid autoantibodies or hormones. The frequency of circulating CD4^+^CXCR5^+^ICOS^high^ Tfh cells was positively correlated with the level of TgAb (Figure 3(a)) but not correlated with the levels of thyroid hormones (Supplementary Figure 3A). However, the frequency of circulating CD4^+^CXCR5^+^PD-1^+^ Tfh cells was not correlated with any thyroid autoantibodies or hormones (Supplementary Figures 3B and 3C).

Compared to circulating Tfh cells, there was no significant correlation between the frequency of intrathyroidal CD4^+^CXCR5^+^ICOS^high^ Tfh cells or the levels of autoantibodies in GD patients (Supplementary Figure 3D). The frequency of intrathyroidal CD4^+^CXCR5^+^ICOS^high^ Tfh cells was negatively correlated with the concentration of FT3 in GD patients (Figure 3(b)). Additionally, the frequency of infiltrating CD4^+^CXCR5^+^PD-1^+^ Tfh cells was positively correlated with TgAb levels (Figure 3(c)). Similarly, we found no significant correlation between infiltrating CD4^+^CXCR5^+^PD-1^+^ Tfh cells and thyroid hormones in GD patients (Supplementary Figure 3E).

### 3.4. The Frequency of Tfh Cells Is Associated with the Levels of TPOAb, TgAb, and FT3 in HT Patients

**Figure 4:**
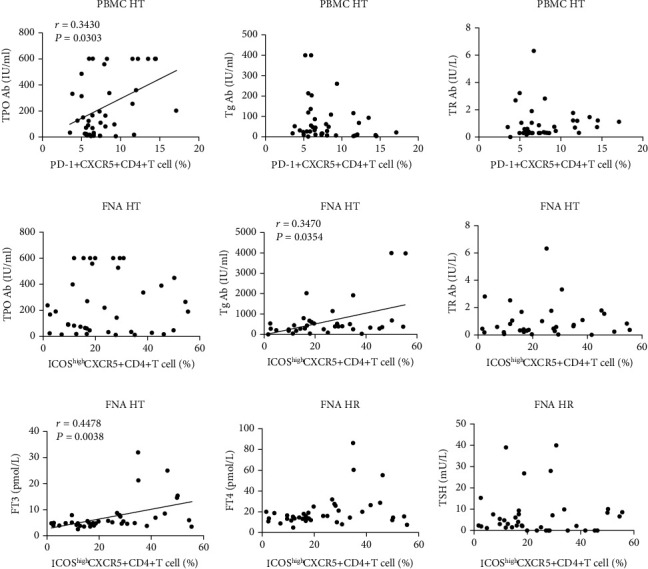
Correlations between Tfh cells and autoantibodies or thyroid function in HT patients. (a) Correlation between the frequency of circulating CD4^+^CXCR5^+^PD-1^+^ Tfh cells and the levels of autoantibodies in HT patients. (b) Correlation between the frequency of intrathyroidal CD4^+^CXCR5^+^ICOS^high^ Tfh cells and the levels of autoantibodies in HT patients. (c) Correlation between the frequency of intrathyroidal CD4^+^CXCR5^+^ICOS^-high^ Tfh cells and the levels of FT3, FT4, and TSH in HT patients.

In HT patients, the frequency of circulating CD4^+^CXCR5^+^PD-1^+^ Tfh cells was only significantly associated with TPOAb levels (Figure 4(a)). However, we found no association between circulating Tfh cells and autoantibodies or hormones (Supplementary Figures 4A–4C). In HT FNA samples, there was a significant positive correlation between the frequency of intrathyroidal CD4^+^CXCR5^+^ICOS^high^ Tfh cells and serum TgAb or FT3 levels (Figures 4(b) and 4(c)). In contrast, no significant correlation was identified between the frequency of intrathyroidal CD4^+^CXCR5^+^PD-1^+^ Tfh cells and the levels of autoantibodies or thyroid hormones in HT patients (Supplementary Figures 4D and 4E).

### 3.5. The Frequency of Intrathyroidal Tfh Cells Varied in AITD Patients with Different Disease Durations

**Figure 5:**
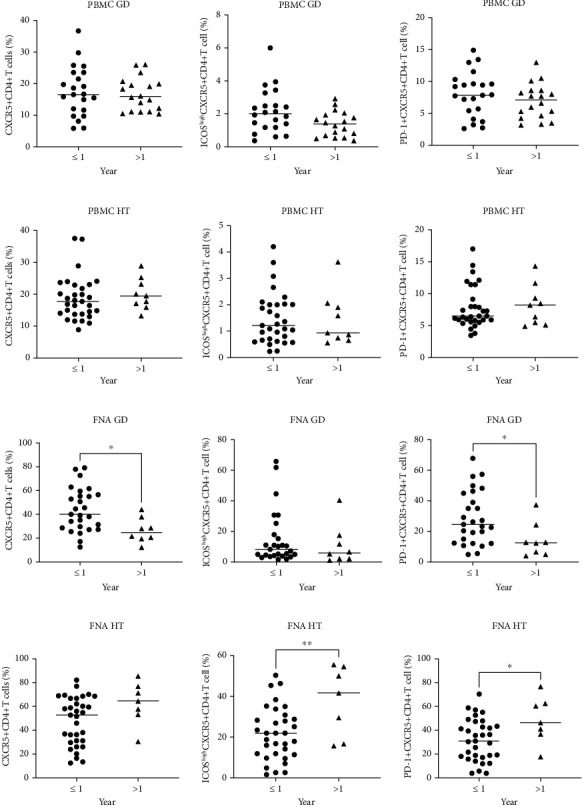
The frequency of Tfh cells in GD and HT patients with different disease durations. The frequencies of circulating CD4^+^CXCR5^+^, CD4^+^CXCR5^+^ICOS^high^, and CD4^+^CXCR5^+^PD-1^+^ Tfh cells in GD (a) and HT (b) patients diagnosed within 1 year and in those with a disease duration > 1 year. The frequencies of intrathyroidal CD4^+^CXCR5^+^, CD4^+^CXCR5^+^ICOS^high^, and CD4^+^CXCR5^+^PD-1^+^ Tfh cells in GD (c) and HT (d) patients diagnosed within 1 year and in those with a disease duration > 1 year.

According to the disease duration, we divided the AITD patients into two groups: patients diagnosed within 1 year (≤1 year) and those diagnosed more than 1 year (>1 year). No significant difference in the frequency of circulating Tfh cells was observed between the two groups in either GD or HT patients (Figures 5(a) and 5(b)). Moreover, there was a significant decrease in the frequencies of intrathyroidal CD4^+^CXCR5^+^ T cells and CD4^+^CXCR5^+^PD-1^+^ Tfh cells in GD patients with a longer disease duration (>1 year) compared with those diagnosed within 1 year (*P* = 0.0157, *P* = 0.0345) (Figure 5(c)). However, in HT patients, the frequencies of intrathyroidal CD4^+^CXCR5^+^ICOS^high^ and CD4^+^CXCR5^+^PD-1^+^ T cells were significantly elevated in patients with a longer disease duration (>1 year) compared with those newly diagnosed (≤1 year) (*P* = 0.0081, *P* = 0.0277) (Figure 5(d)).

In AITD patients, some GD patients were treated with antithyroid drugs (methimazole or propylthiouracil), and some HT patients were supplemented with levothyroxine. We further investigated the effect of drug administration on Tfh cells, but neither antithyroid drugs nor thyroid hormone supplementation had a significant effect on the percentage of Tfh cells in AITD patients (Supplementary Figure 5).

### 3.6. Circulating and Thyroid-Infiltrating Tfh Cells in AITD

**Figure 6:**
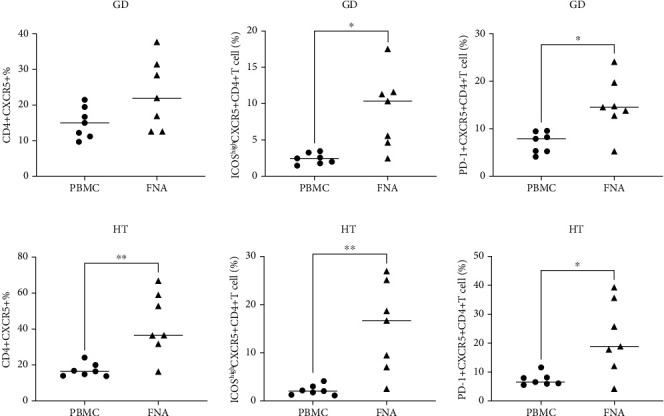
The frequency of Tfh cells in the circulation and thyroid in GD and HT patients. (a) The frequencies of CD4^+^CXCR5^+^, CD4^+^CXCR5^+^ICOS^high^, and CD4^+^CXCR5^+^PD-1^+^ Tfh cells in circulation and thyroid tissue in GD patients. (b) The frequencies of CD4^+^CXCR5^+^, CD4^+^CXCR5^+^ICOS^high^, and CD4^+^CXCR5^+^PD-1^+^ Tfh cells in circulation and thyroid tissue in HT patients.

We measured the levels of both circulating and infiltrating Tfh cells in seven GD and seven HT patients. There was a significant increase in CD4^+^CXCR5^+^ICOS^high^ and CD4^+^CXCR5^+^PD-1^+^ Tfh cells in FNA samples from GD patients (*P* = 0.0160, *P* = 0.0104) (Figure 6(a)). Similarly, Tfh cell levels were higher in the thyroid tissue than in the peripheral circulation in HT patients (*P* = 0.0057, *P* = 0.0078, and *P* = 0.0173) (Figure 6(b)). However, possibly due to the limited number of cases, we did not find a significant correlation between circulation and intrathyroidal Tfh (Supplementary Figures 6A and 6B).

## 4. Discussion

Although GD and HT have distinct clinical manifestations, they are both characterized by thyroid autoantibodies and autoreactive lymphocytes in thyroid tissues. Dysregulation of Tfh activities can contribute to pathogenic autoantibody production. Tfh cells can play an important role in the promotion of autoimmune diseases [25, 26] as well as in the pathogenesis of AITD. In this study, we provided for the first time a comprehensive view of Tfh cells and demonstrated the differences in Tfh cells between GD and HT patients in both peripheral blood and intrathyroidal samples.

Previously, studies have shown that the frequency of activated circulating Tfh cells is increased in SLE patients [25, 27]. In addition, Tfh cells are correlated with the diversity and titers of autoantibodies and with the severity of end-organ involvement. However, the correlations between Tfh cells and disease activity status or treatment are not concordant [28, 29]. Zhu et al. [20] previously showed that the frequency of Tfh cells was increased in the peripheral blood of AITD patients and described a close correlation between the percentages of circulating CD4^+^CXCR5^+^ICOS^high^ T cells and FT3 or FT4 in GD patients. Our study targeted AITD and investigated Tfh cells in peripheral blood and thyroid tissues.

Conversely, we demonstrated that the frequency of Tfh cells was higher in thyroid tissue and that intrathyroidal Tfh cells were more relevant to thyroid function. Based on a previous study, the clinical features of the subject, especially the difference in baseline thyroid function, may explain the different correlations between thyroid function and Tfh cells. The typical clinical manifestations of GD and HT are reversed. Since our study is cross-sectional, we did not follow up patients to observe the changes in Tfh of AITD patients as the disease progressed.

According to the disease duration, we divided the AITD patients into two groups (patients diagnosed within 1 year and more than 1 year) and compared the frequency of Tfh cells. Interestingly, we found that the levels of infiltrating Tfh cells in the thyroid showed different correlations with the progression of AITD. Intrathyroidal Tfh cells decreased with the progression of GD but were significantly increased in HT patients with longer disease durations, which suggested that the dysregulation of Tfh cells may play a different role in the pathogenesis of GD and HT. Although the formation of Tfh cells requires both BCL6 and ICOS, circulating Tfh cells do not express BCL6 [27, 30, 31]. Therefore, we did not examine BCL6 in our study.

Consistent with our data, it was previously reported that circulating Tfh cells do not express high amounts of CXCR5 or PD-1 [32]. The difference between HT and GD is the extent of the dysregulated reaction of the immune system [23]. Interestingly, we found that intrathyroidal CD4^+^CXCR5^+^ICOS^high^ Tfh cells were more positively associated with HT patients than GD patients. Inducible T cell costimulator (ICOS) is an activating costimulatory immune checkpoint expressed in activated T cells. Its ligand, ICOSL, is expressed in antigen-presenting cells and somatic cells. The expression of ICOS and ICOSL is linked to the release of cytokines induced by activation of the immune response. ICOS is a conserved mediator of immune responses across multiple immunotherapy strategies [33]. Recent studies highlighted the importance of ICOS signaling in promoting CXCR5 expression and possibly the generation of Tfh cells [34]. Thus, modulation of ICOS signaling has the potential to mitigate disease severity in some human autoimmune disorders [35, 36]. The secretion of cytokines could cause apoptosis of infiltrating lymphocytes and the proliferation of thyrocytes, further resulting in GD. In HT, increased levels of cytokines could cause apoptosis of thyrocytes but not infiltration of lymphocytes [23]. Therefore, apoptotic death of thyrocytes may induce more CD4^+^CXCR5^+^ICOS^high^ Tfh cells to converge to the thyroid tissue in HT.

In this paper, we examined Tfh cells in both peripheral blood and thyroid tissues. We demonstrated that the frequencies of Tfh cells were higher in the thyroid. Tfh cells appeared to control antigen-specific antibody responses, especially targeting Tg self-antigens. Furthermore, intrathyroidal Tfh cells were closely associated with the progression of AITD. In summary, intrathyroidal Tfh cells might play a role in the pathogenesis of AITD and are potential immunobiomarkers for AITD.

## Figures and Tables

**Table 1:** Baseline characteristics of subjects with peripheral blood samples.

	Healthy controls	GD	HT	*P* value
Number	40	40	40	—
Age (year)	31 (27, 37)	35 (26, 48)	42 (29, 51)	0.1207
Female (%)	26 (65.0)	32 (80.0)	34 (85.0)	0.0887
Duration (months)	—	4 (0, 57)	0 (0, 11)	0.0102
Medication (%)	—	17 (42.5)	20 (50.0)	0.6541
Dosage				
Methimazole (mg)	—	13 (8, 15)	—	—
Levothyroxine (*μ*g)	—	—	50 (25, 50)	—
FT3 (pmol/L)	4.87 (4.18, 5.27)	14.21 (6.31, 23.99)^a^	4.57 (4.18, 5.01)^c^	<0.0001
FT4 (pmol/L)	17.65 (15.73, 19.18)	42.35 (22.63, 69.34)^a^	15.93 (13.68, 18.25)^c^	<0.0001
TSH (mIU/L)	2.210 (1.220, 3.173)	0.005 (0.005, 0.005)^a^	2.810 (1.785, 5.853)^c^	<0.0001
TPOAb (IU/mL)	11.55 (8.38, 15.10)	244.15 (30.53, 502.45)^a^	138.00 (26.48, 454.80)^a^	<0.0001
TgAb (IU/mL)	16.30 (13.78, 19.05)	94.35 (11.38, 408.35)^b^	300.90 (96.10, 854.30)^a,d^	<0.0001
TRAb (IU/L)	0.80 (0.80, 0.80)	9.02 (5.53, 17.75)^a^	0.58 (0.30, 1.19)^c^	<0.0001

Data are presented as the number (%) or median (P25, P75). GD: Graves' disease; HT: Hashimoto's thyroiditis; FT3: free T3; FT4: free T4; TSH: thyroid-stimulating hormone; TGAb: thyroglobulin antibody; TPOAb: thyroid peroxidase antibodies; TRAb: thyrotropin receptor antibody. ^a^Compared with healthy control subjects *P* < 0.0001. ^b^Compared with healthy control subjects *P* < 0.01. ^c^Compared with Graves' disease subjects *P* < 0.0001. ^d^Compared with Graves' disease subjects *P* < 0.01.

**Table 2:** Baseline characteristics of subjects with thyroid fine-needle aspiration samples.

	nAITD	GD	HT	*P* value
Number	22	35	40	—
Age (year)	41 (30, 50)	34 (26, 46)	36 (27, 46)	0.3518
Female (%)	15 (68.2)	26 (74.3)	34 (85.0)	0.2757
Duration (months)	1 (0, 8)	1 (0, 12)	1 (0, 5)	0.4226
Medication (%)	—	18 (51.4)	10 (25.0)	0.0305
Dosage				—
Methimazole (mg)	—	20 (15, 30)	—	—
Levothyroxine (*μ*g)	—	—	25 (25, 50)	—
FT3 (pmol/L)	4.85 (4.60, 5.14)	17.45 (10.70, 30.75)^a^	4.85 (4.17, 5.89)^b^	<0.0001
FT4 (pmol/L)	16.02 (15.01, 17.28)	46.88 (35.83, 77.51)^a^	15.42 (12.02, 20.69)^b^	<0.0001
TSH (mIU/L)	1.940 (1.268, 2.315)	0.005 (0.005, 0.006)^a^	2.845 (0.416, 8.310)^b^	<0.0001
TPOAb (IU/mL)	12.90 (10.30, 18.00)	280.90 (107.95, 571.20)^a^	154.40 (31.60, 434.43)^a^	<0.0001
TgAb (IU/mL)	12.25 (10.00, 18.35)	265.80 (40.08, 449.53)^a^	380.90 (252.85, 580.85)^a^	<0.0001
TRAb (IU/L)	0.31 (0.30, 0.80)	6.14 (2.89, 17.31)^a^	0.55 (0.25, 1.31)^c^	<0.0001

Data are presented as the number (%) or median (P25, P75). nAITD: nonautoimmune thyroid disease; GD: Graves' disease; HT: Hashimoto's thyroiditis; FT3: free T3; FT4: free T4; TSH: thyroid-stimulating hormone; TGAb: thyroglobulin antibody; TPOAb: thyroid peroxidase antibodies; TRAb: thyrotropin receptor antibody. ^a^Compared with nAITD control subjects *P* < 0.0001. ^b^Compared with Graves' disease subjects *P* < 0.0001. ^c^Compared with Graves' disease subjects *P* < 0.001.

## Data Availability

The data used to support the findings of this study are included within the article. The data used to support the findings of this study are included within the supplementary information file(s).

## Supplementary Materials

Supplementary Materials Supplementary Figure 1: correlations between circulating CD4^+^CXCR5^+^ T cells and autoantibodies or thyroid function in GD and HT patients. Correlation between the frequency of circulating CD4^+^CXCR5^+^ cells and the levels of autoantibodies (A) or thyroid function (B) in GD patients. Correlation between the frequency of circulating CD4^+^CXCR5^+^ cells and the levels of autoantibodies (C) or thyroid function (D) in HT patients. Supplementary Figure 2: correlations between intrathyroidal CD4^+^CXCR5^+^ T cells and autoantibodies or thyroid function in GD and HT patients. Correlation between the frequency of intrathyroidal CD4^+^CXCR5^+^ cells and the levels of autoantibodies (A) or thyroid function (B) in GD patients. Correlation between the frequency of intrathyroidal CD4^+^CXCR5^+^ cells and the levels of autoantibodies (C) or thyroid function (D) in HT patients. Supplementary Figure 3: correlations between Tfh cells and autoantibodies or thyroid function in GD patients. (A) Correlation between the frequency of circulating CD4^+^CXCR5^+^ICOS^high^ Tfh cells and the levels of FT3, FT4, and TSH in GD patients. (B) Correlation between the frequency of circulating CD4^+^CXCR5^+^PD-1^+^ Tfh cells and the levels of autoantibodies in GD patients. (C) Correlation between the frequency of circulating CD4^+^CXCR5^+^PD-1^+^ Tfh cells and the levels of FT3, FT4, and TSH in GD patients. (D) Correlation between the frequency of intrathyroidal CD4^+^CXCR5^+^ICOS^high^ Tfh cells and the levels of autoantibodies in GD patients. (E) Correlation between the frequency of intrathyroidal CD4^+^CXCR5^+^PD-1^+^ Tfh cells and the levels of FT3, FT4, and TSH in GD patients. Supplementary Figure 4: correlations between Tfh cells and autoantibodies or thyroid function in HT patients. (A) Correlation between the frequency of circulating CD4^+^CXCR5^+^ICOS^high^ Tfh cells and the levels of autoantibodies in HT patients. (B) Correlation between the frequency of circulating CD4^+^CXCR5^+^ICOS^high^ Tfh cells and the levels of FT3, FT4, and TSH in HT patients. (C) Correlation between the frequency of circulating CD4^+^CXCR5^+^PD-1^+^ Tfh cells and the levels of FT3, FT4, and TSH in HT patients. (D) Correlation between the frequency of intrathyroidal CD4^+^CXCR5^+^PD-1^+^ Tfh cells and the levels of autoantibodies in HT patients. (E) Correlation between the frequency of intrathyroidal CD4^+^CXCR5^+^PD-1^+^ Tfh cells and the levels of FT3, FT4, and TSH in HT patients. Supplementary Figure 5: the frequency changes of Tfh cells in GD and HT patients with the therapy. The frequencies of circulating CD4^+^CXCR5^+^, CD4^+^CXCR5^+^ICOS^high^, and CD4^+^CXCR5^+^PD-1^+^ Tfh cells were analyzed in GD (A) and HT (B) patients who undertook antithyroid drug therapy or thyroid hormone supplementation. The frequencies of intrathyroidal CD4^+^CXCR5^+^, CD4^+^CXCR5^+^ICOS^high^, and CD4^+^CXCR5^+^PD-1^+^ Tfh cells were analyzed in GD (C) and HT (D) patients who undertook antithyroid drug therapy or thyroid hormone supplementation. Supplementary Figure 6: correlations between circulating and intrathyroidal Tfh cells in GD and HT patients. (A) Correlation between the circulating and intrathyroidal Tfh cells in GD patients. (B) Correlation between the circulating and intrathyroidal Tfh cells in HT patients.
