# Supplementary material for: The Frequency of Intrathyroidal Follicular Helper T Cells Varies with the Progression of Graves' Disease and Hashimoto's Thyroiditis
Source: J Immunol Res. 2022 Feb 2;2022:4075522. doi: 10.1155/2022/4075522 (PMC8872690; doi:10.1155/2022/4075522)
Supplement: Supplementary Materials — Supplementary Figure 1: correlations between circulating CD4+CXCR5+ T cells and autoantibodies or thyroid function in GD and HT patients. Correlation between the frequency of circulating CD4+CXCR5+ cells and the levels of autoantibodies (A) or thyroid function (B) in GD patients. Correlation between the frequency of circulating CD4+CXCR5+ cells and the levels of autoantibodies (C) or thyroid function (D) in HT patients. Supplementary Figure 2: correlations between intrathyroidal CD4+CXCR5+ T cells and autoantibodies or thyroid function in GD and HT patients. Correlation between the frequency of intrathyroidal CD4+CXCR5+ cells and the levels of autoantibodies (A) or thyroid function (B) in GD patients. Correlation between the frequency of intrathyroidal CD4+CXCR5+ cells and the levels of autoantibodies (C) or thyroid function (D) in HT patients. Supplementary Figure 3: correlations between Tfh cells and autoantibodies or thyroid function in GD patients. (A) Correlation between the frequency of circulating CD4+CXCR5+ICOShigh Tfh cells and the levels of FT3, FT4, and TSH in GD patients. (B) Correlation between the frequency of circulating CD4+CXCR5+PD-1+ Tfh cells and the levels of autoantibodies in GD patients. (C) Correlation between the frequency of circulating CD4+CXCR5+PD-1+ Tfh cells and the levels of FT3, FT4, and TSH in GD patients. (D) Correlation between the frequency of intrathyroidal CD4+CXCR5+ICOShigh Tfh cells and the levels of autoantibodies in GD patients. (E) Correlation between the frequency of intrathyroidal CD4+CXCR5+PD-1+ Tfh cells and the levels of FT3, FT4, and TSH in GD patients. Supplementary Figure 4: correlations between Tfh cells and autoantibodies or thyroid function in HT patients. (A) Correlation between the frequency of circulating CD4+CXCR5+ICOShigh Tfh cells and the levels of autoantibodies in HT patients. (B) Correlation between the frequency of circulating CD4+CXCR5+ICOShigh Tfh cells and the levels of FT3, FT4, and TSH in HT patient [file 4075522.f1.pdf]

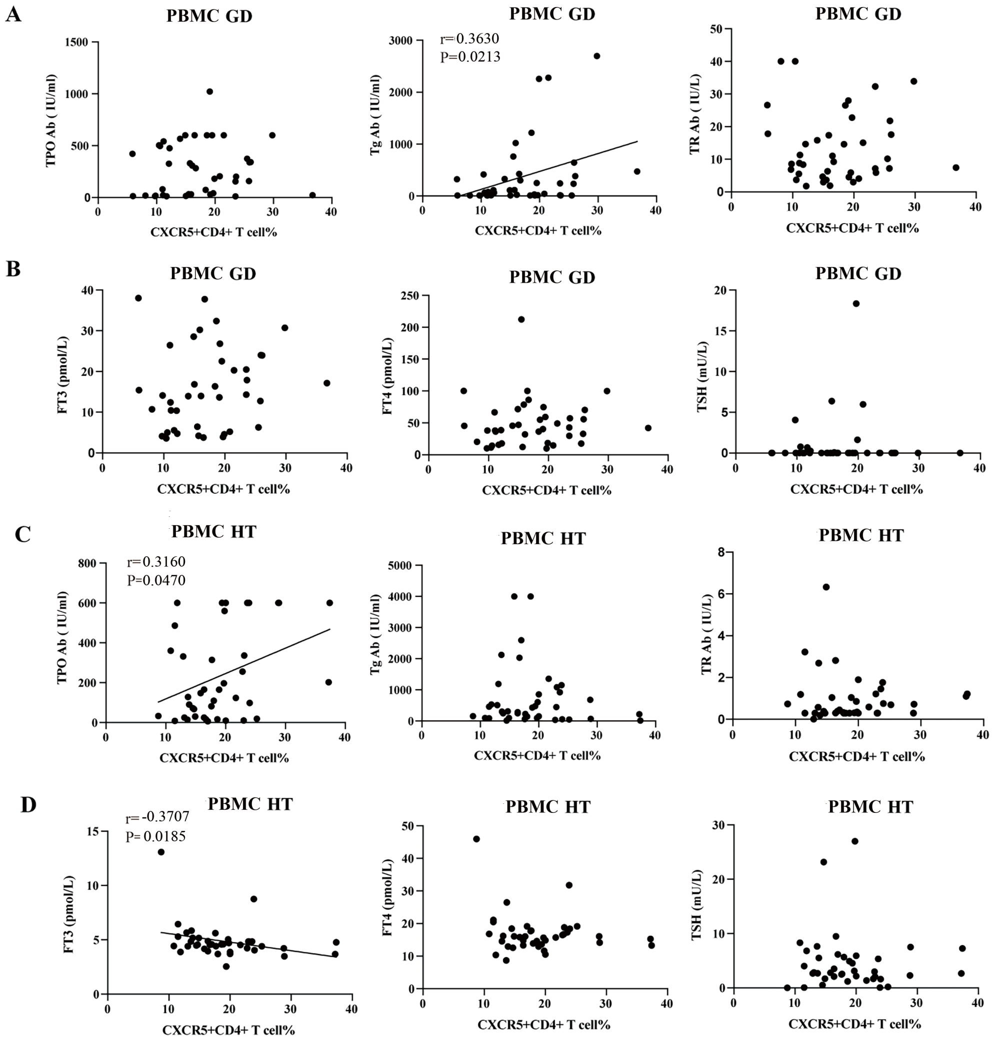

Supplementary Figure 1. Correlations between circulating $CD4^+CXCR5^+$ T cells and autoantibodies or thyroid function in GD and HT patients. Correlation between the frequency of circulating $CD4^+CXCR5^+$ cells and the levels of autoantibodies(A) or thyroid function(B) in GD patients. Correlation between the frequency of circulating $CD4^+CXCR5^+$ cells and the levels of autoantibodies(C) or thyroid function(D) in HT patients.

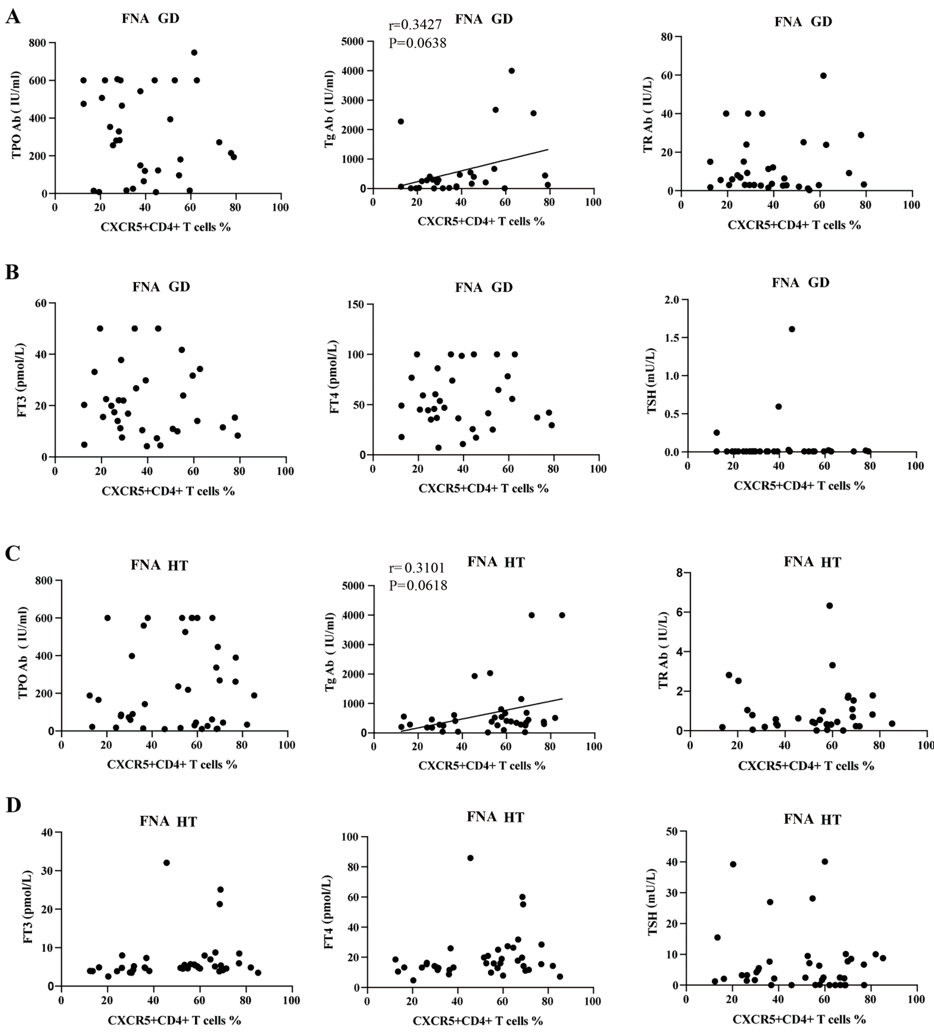

Supplementary Figure 2. Correlations between intrathyroidal CD4$^+$CXCR5$^+$ T cells and autoantibodies or thyroid function in GD and HT patients. Correlation between the frequency of intrathyroidal CD4$^+$CXCR5$^+$ cells and the levels of autoantibodies(A) or thyroid function(B) in GD patients. Correlation between the frequency of intrathyroidal CD4$^+$CXCR5$^+$ cells and the levels of autoantibodies(C) or thyroid function(D) in HT patients.

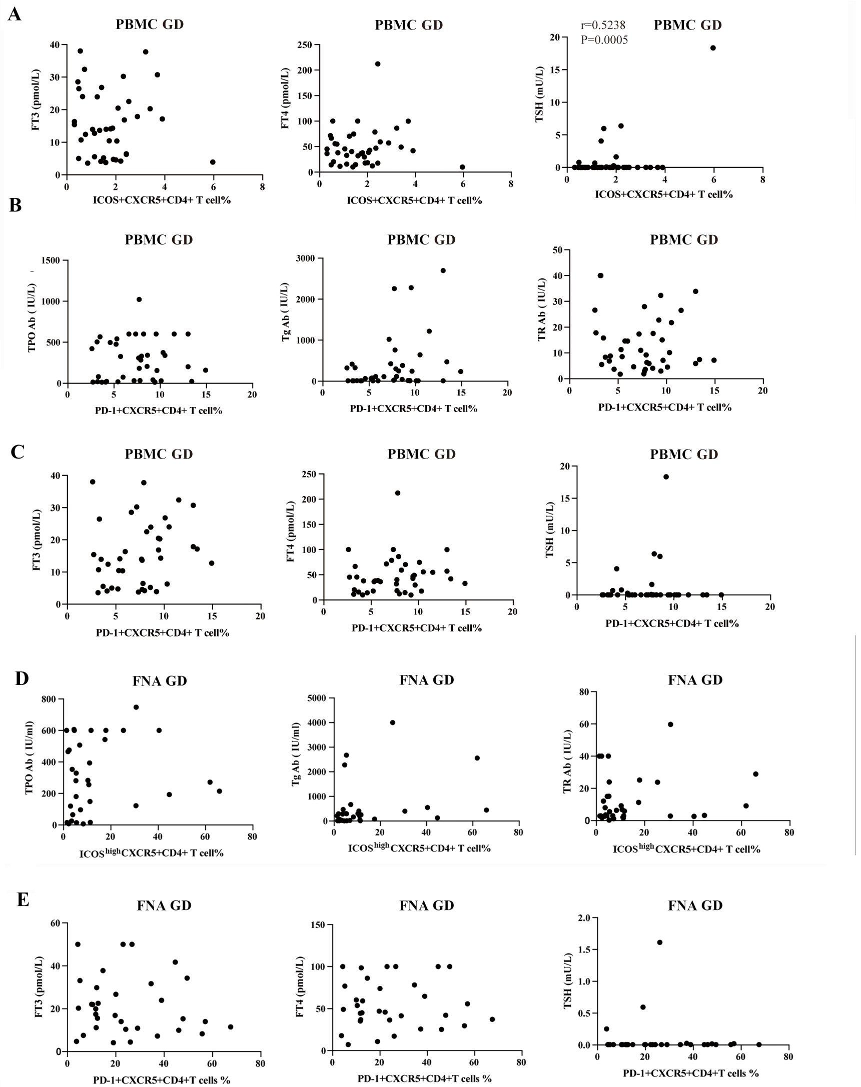

Supplementary Figure 3. Correlations between Tfh cells and autoantibodies or thyroid function in GD patients. (A)Correlation between the frequency of circulating $CD4^+CXCR5^+$ $ICOS^{high}$ Tfh cells and the levels of FT3, FT4 and TSH in GD patients. (B)Correlation between the frequency of circulating $CD4^+CXCR5^+$ PD-1^+ Tfh cells and the levels of autoantibodies in GD patients. (C)Correlation between the frequency of circulating $CD4^+CXCR5^+$ PD-1^+ Tfh cells and the levels of FT3, FT4 and TSH in GD patients. (D)Correlation between the frequency of intrathyroidal $CD4^+CXCR5^+$ $ICOS^{high}$ Tfh cells and the levels of autoantibodies in GD patients. (E) Correlation between the frequency of intrathyroidal $CD4^+CXCR5^+$ PD-1^+ Tfh cells and the levels of FT3, FT4 and TSH in GD patients.

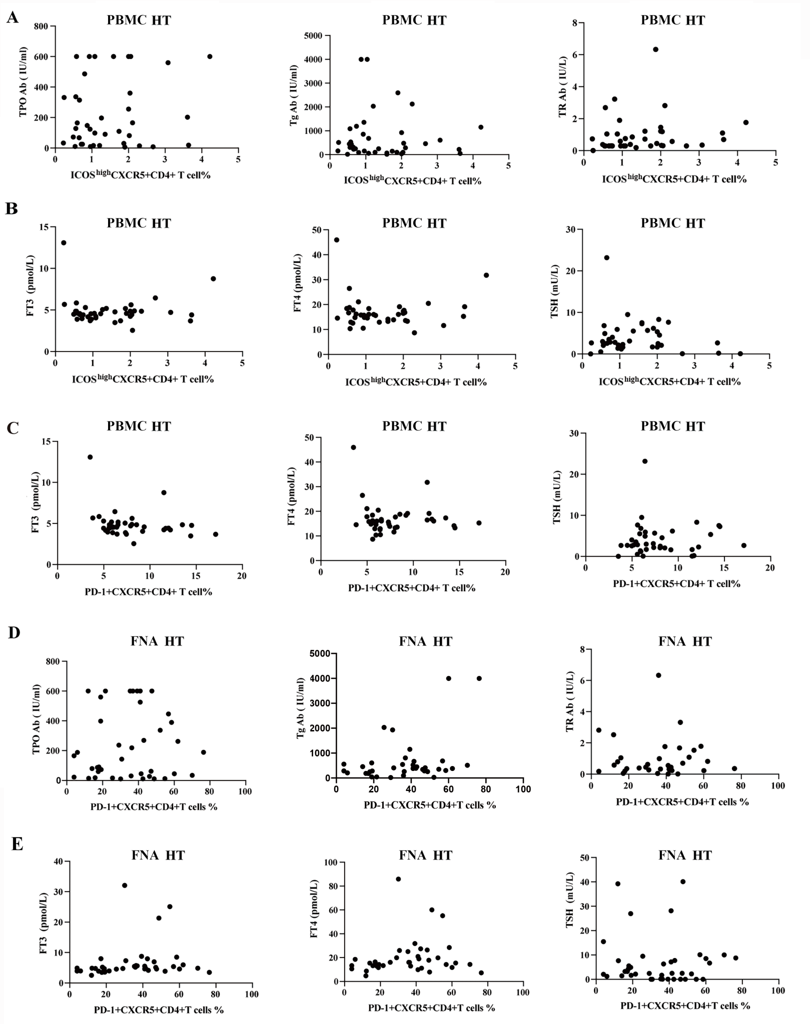

Supplementary Figure 4. Correlations between Tfh cells and autoantibodies or thyroid function in HT patients. (A)Correlation between the frequency of circulating $CD4^{+}CXCR5^{+}$ ICOS^{high} Tfh cells and the levels of autoantibodies in HT patients. (B)Correlation between the frequency of circulating $CD4^{+}CXCR5^{+}$ ICOS^{high} Tfh cells and the levels of FT3, FT4 and TSH in HT patients. (C)Correlation between the frequency of circulating $CD4^{+}CXCR5^{+}$ PD-1^{+} Tfh cells and the levels of FT3, FT4 and TSH in HT patients. (D)Correlation between the frequency of intrathyroidal $CD4^{+}CXCR5^{+}$ PD-1^{+} Tfh cells and the levels of autoantibodies in HT patients. (E) Correlation between the frequency of intrathyroidal $CD4^{+}CXCR5^{+}$ PD-1^{+} Tfh cells and the levels of FT3, FT4 and TSH in HT patients.

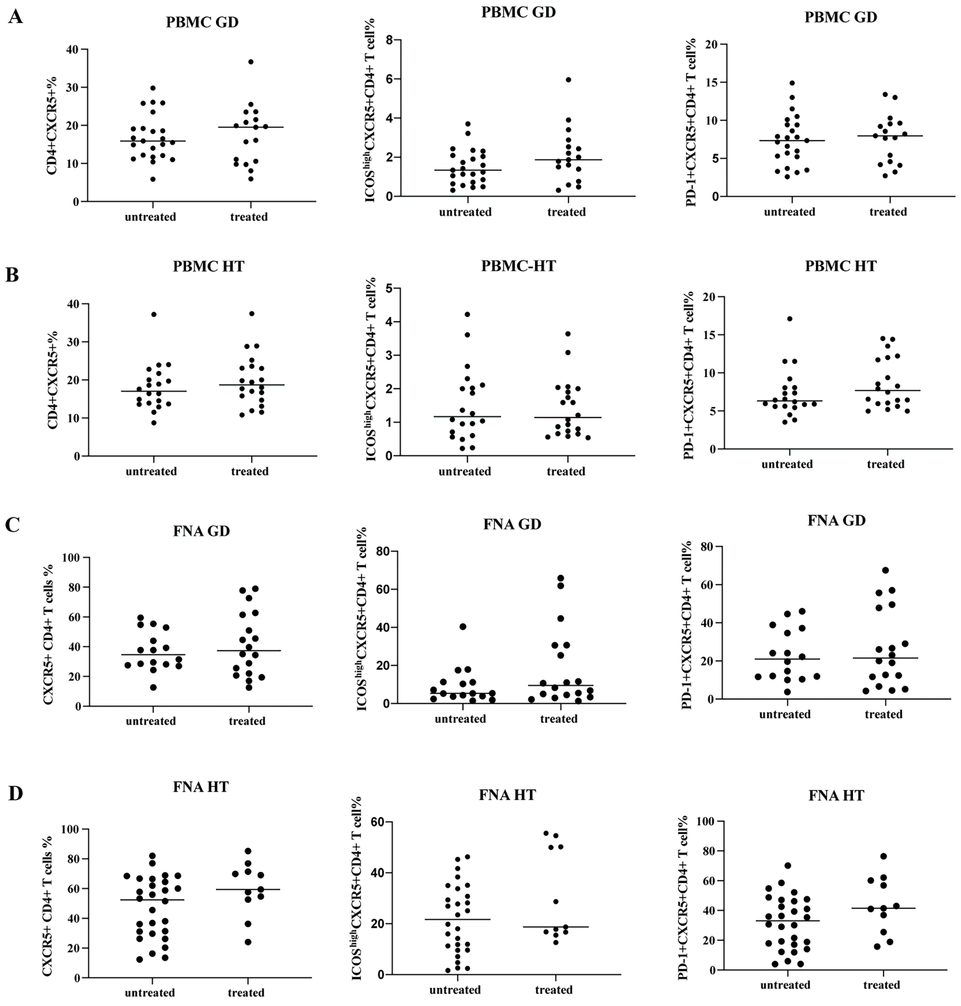

Supplementary Figure 5. The frequency changes of Tfh cells in GD and HT patients with the therapy. The frequencies of circulating CD4^{+}CXCR5^{+}, CD4^{+}CXCR5^{+} ICOS ^{high} and CD4^{+}CXCR5^{+} PD-1^{+} Tfh cells were analyzed in GD (A) and HT(B) patients who were undertook anti-thyroid drugs therapy or thyroid hormone supplementation. The frequencies of intrathyroidal CD4^{+}CXCR5^{+}, CD4^{+}CXCR5^{+} ICOS^{high} and CD4^{+}CXCR5^{+} PD-1^{+} Tfh cells were analyzed in GD (C) and HT(D) patients who were undertook anti-thyroid drugs therapy or thyroid hormone supplementation.

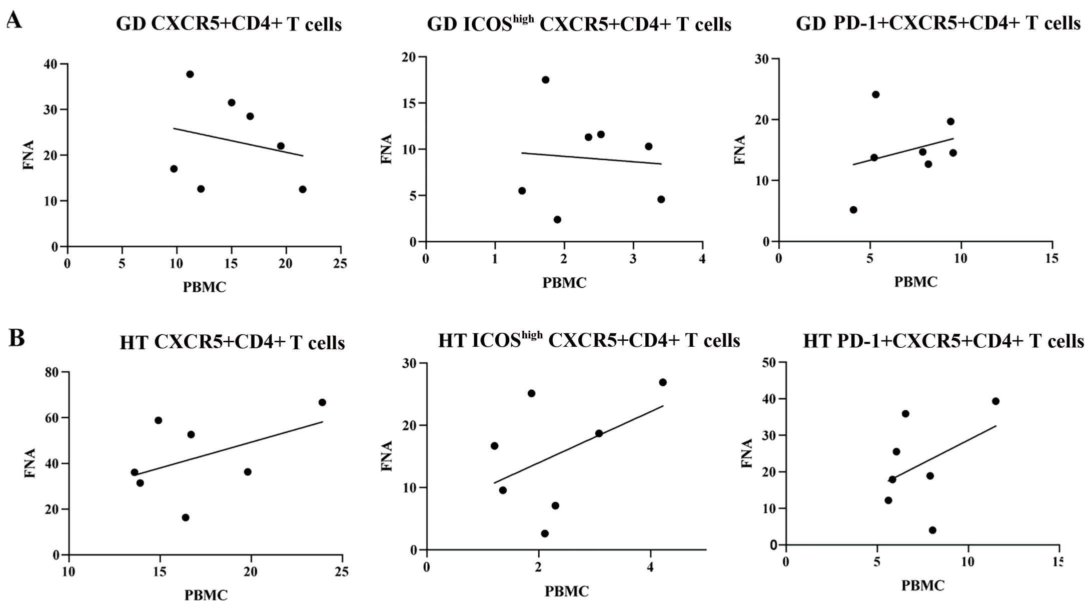

Supplementary Figure 6. Correlations between circulating and intrathyroidal Tfh cells in GD and HT patients. (A)Correlation between the circulating and intrathyroidal Tfh cells in GD patients. (B)Correlation between the circulating and intrathyroidal Tfh cells in HT patients.
